# Inhalation Exposure to Gaseous and Particulate Bound Mercury Present in the Ambient Air over the Polluted Area of Southern Poland

**DOI:** 10.3390/ijerph17144999

**Published:** 2020-07-11

**Authors:** Halina Pyta, Kamila Widziewicz-Rzońca, Krzysztof Słaby

**Affiliations:** Institute of Environmental Engineering Polish Academy of Sciences, M. Skłodowskiej-Curie 34 St., 41-819 Zabrze, Poland; halina.pyta@ipis.zabrze.pl (H.P.); krzysztof.slaby@ipis.zabrze.pl (K.S.)

**Keywords:** mercury, TGM, PBM, PM_2.5_, urban background, rural background, inhalation toxicity, exposure rate

## Abstract

This study concerns the concentrations of gaseous and particle-bound mercury present in ambient air of two Polish sites, differing in terms of emission structure, and the estimation of inhalation risks related to those Hg species. The measurements of total gaseous mercury (TGM) and PM_2.5_-bound mercury (PBM) were performed at an urban station in Zabrze and a rural station in Złoty Potok, in 2014–2015. Both sites are located in Silesia, considered one of the European air pollution hot-spots. TGM was measured on-line (Tekran 2537). PM_2.5_ samples were taken with the use of low volume samplers. Hg contents in PM were determined by the CVAAS method following thermal decomposition. The median concentrations of TGM and PBM in Zabrze were 2.48 ng m^−3^ and 37.87 pg m^−3^, respectively; meanwhile in Zloty Potok, these were 1.69 ng m^−3^ and 27.82 pg m^−3^, respectively. Clearly, seasonal variability of TGM and PBM concentrations were observed, reflecting the importance of Hg and PM emissions from coal combustion for power and heating purposes. Health risk assessment was performed using a deterministic approach by the most conservative exposure scenario. The obtained HQ ratios and the cumulative HI indexes were below the limit value (<1). This means an unlikely health hazard due mercury inhalation.

## 1. Introduction

Mercury (Hg) is a naturally occurring element found in the environment in inorganic form (I Hg; elemental mercury Hg^0^, in liquid and gaseous state, and as numerous mercury salts, e.g., mercuric chloride HgCl_2_), and organic compounds (mostly short-chain alkyl derivatives, e.g., methylmercury MeHg). I Hg can undergo methylation by biota mainly in aquatic environment and then bioaccumulation and biomagnification in food webs. Mercury is persistent in the environment. It cycles among the air, ocean, land, and biosphere [1]. Once released to the atmosphere, it can be transported over long distances and deposited on the Earth’s surface even in remote areas, leading to contamination of aquatic and terrestrial ecosystems [2]. Although the atmosphere is a small reservoir of Hg, relative to oceans and lands, it is viewed as the main pathway for the global cycling of mercury. Atmospheric Hg is currently measured as three operationally defined forms: gaseous elemental Hg (Hg^0^, GEM), gaseous oxidized Hg (GOM), with the sum of GEM and GOM designated as total gaseous mercury (TGM), and particulate bound Hg (PBM). The main form of atmospheric Hg is relatively chemically inert Hg^0^, which can remain in the air for up to a year [3]. Hg^0^ generally comprises more than 95% of total airborne Hg [4,5] and is fairly uniformly distributed in the Northern Hemisphere, with a range of concentrations of 1.3 v–1.7 ng m^−3^ [6]. The concentrations of GOM and PBM (usually expressed in pg m^−3^) are much lower than Hg^0^, they are essential for removing of mercury from the air by wet and dry deposition [5]. All Hg forms can derive from a multitude of natural and anthropogenic sources, both primary and secondary [7,8]. The latest estimates on Hg emissions of natural origin, including primary processes and re-emissions, were assessed to be 5207 Mg yr^−1^ [9]. The major anthropogenic sources of mercury, on a global scale, are artisanal and small-scale gold mining, combustion of fossil fuels for power and heat generation, industry of non-ferrous metals, cement production and large-scale gold production [10]. According to the AMAP/UNEP inventory, the global anthropogenic emission has been estimated to 1960 Mg yr^−1^ [8] and is a main factor disrupting the natural Hg cycling.

Mercury is considered as one of the top 10 chemicals of major public health concern by the World Health Organization (WHO) [11,12]. Toxicity of this element varies with its chemical form and the dose, as well as the route of exposure, the exposure duration, and individual vulnerability of the person exposed [13]. For mercury, the most sensitive subpopulations are developing organisms of the fetus, the newborn, and young children [12].

The respiratory tract is the main absorption route for both atmospheric mercury forms: TGM (mainly Hg^0^) and PBM (mainly Hg^2+^ and Hg^+^). Exposure to PBM may partly occur through the gastrointestinal system. Only 0.01% of Hg^0^ that reaches the gastrointestinal tract is absorbed, because of its conversion to Hg^2+^ and binding to sulfhydryl groups [7]. As much as 85% of the inhaled dose of Hg^0^ can be absorbed into the bloodstream and then distributed throughout the body [11,14]. After oxidation to Hg^2+^ via the hydrogen peroxide-catalase pathway [15], it easily binds to intra-cellular molecules [13,16]. Elemental mercury is toxic to the central and peripheral nervous system. It can cause neurological and behavioral disorders, such as tremors, neuromuscular effects, polyneuropathy, emotional changes, insomnia, memory loss, headaches, and cognitive and motor dysfunction. Long-term exposure to higher Hg^0^ levels (>20 µg m^−3^) may lead to changes in renal function and irritation of the respiratory tract [15]. There are only very few data available on the effects of inhalation of I Hg compounds (Hg^2+^, Hg^+^) [17]. Quantitative information on these effects on humans is essentially not-existant [7]. The available reports suggest that absorption of I Hg may occur after inhalation or ingestion of dusts [16]. It is thought that I Hg absorption via the lungs is low, due to deposition of coarse particles in the upper respiratory tract and subsequent mucociliary clearance [7,18]. However Clarkson [19] reported absorption in dogs to be 40% via inhalation of HgCl_2_. Monovalent mercury compounds have limited solubility, so their absorption is less likely than for hydrophilic divalent forms [18]. Absorbed divalent cation from exposure to Hg^2+^ compounds can be reduced and released as elemental mercury vapor [15]. Inorganic mercury may damage the peripheral nervous system and lead to nephrotic syndrome in humans [15]. Boffetta and co-authors [20] found that chronic occupational exposure to I Hg was significantly associated with hypertension, heart diseases other than ischaemic, pneumoconiosis, nephritis, and nephrosis.

The International Agency for Research on Cancer concluded that mercury (CAS No. 7439-97-6) is not classified as carcinogenic to humans (Group 3) [21].

Inhalation exposure to Hg and its compounds is common. However, there is still insufficient information on the potential effects of long-term exposure to atmospheric mercury, depending on its chemical or physical form. Therefore, in this study, we tried to estimate the combined health risk posed by atmospheric mercury in gaseous form (TGM) and bound to PM_2.5_ (respirable fraction of particulates with diameters ≤2.5 µm), based on the methodology recommended by the U.S. Environmental Protection Agency (EPA). The potential health risk from inhalation of the both mercury forms was evaluated by using the hazard quotient (non-cancer outcome). We compared the hazard quotients for two Polish areas (urban and rural background), differing in terms of emission structure and mercury concentrations in the air. The assessment of such risks was done over 2-year measurement campaign (January 2014—December 2015) performed in Silesia Province, the most polluted region in Poland. Poland is the country with the highest Hg emissions in the European Union [22]. Following the Poland’s Informative Inventory Report 2017 [23], these emissions were 9.6 and 10.6 Mg in 2014 and 2015, respectively. In 2015, about 94% of Polish anthropogenic emissions of Hg came from fuel combustion (mostly-hard coal) in heat and power industry (50%), industrial power plants (35%), and residential heating (9%). 

## 2. Study Area

**Figure 1:**
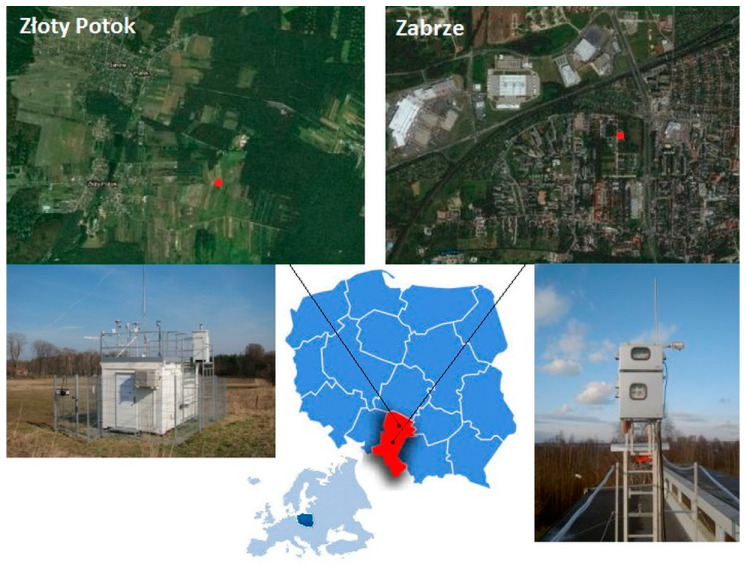
The location of measuring stations in Zabrze and Złoty Potok.

The measurements were conducted at two sampling locations—Zabrze and Złoty Potok—in Silesian Province, a region greatly impacted by the industrial and municipal emissions in Southern Poland (Figure 1). 

**Figure 2:**
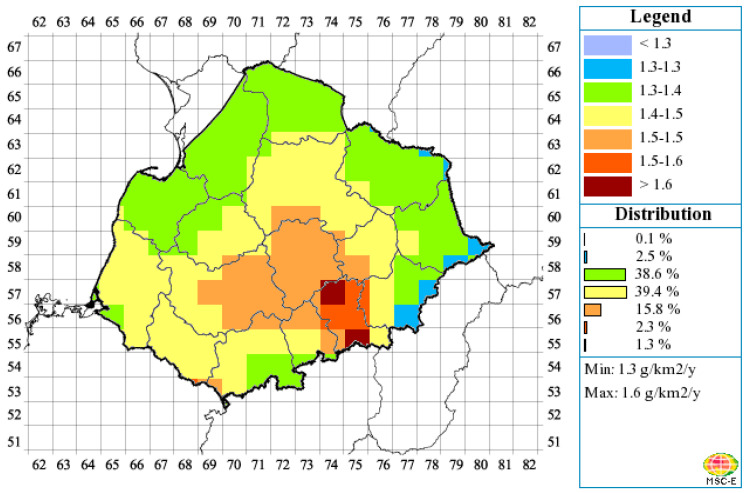
Annual mean concentrations of atmospheric Hg [ng m^−3^] in Poland for 2015 over the EMEP domain (Meteorological Synthesizing Centre-East, 2017; Supplement to EMEP Status Reports 2/2017 and 3/2017 “Heavy metal and POP transboundary pollution in 2015: Concentration and deposition maps, source-receptor matrices, ecosystem-specific deposition and evaluation of model performance”; http://en.msceast.org/index.php/pollution-assessment/emep-domain-menu).

Zabrze (180,000 inhabitants) is one of the 14 cities which together make up the Upper Silesian Agglomeration (2 million inhabitants) in the central part of Silesian Province. Silesia is one of the European air pollution “hot-spots”. This is a region where the risk of high air contamination of mercury occurs as a result of high anthropogenic emissions (Figure 2). Zabrze has the least developed central heating system in the entire agglomeration, most detached houses being individually heated by hard coal combustion. There are two cokeries and several coal fired heat and power plants within the city and in its close vicinity. The measurements were performed in the residential district of Zabrze, within the Institute of Environmental Engineering (IEE). The gaseous mercury sampling point was situated about 14 m above the ground and 1.7 m above the roof of two-storey IEE’ building. Its geographical coordinates (WGS84) were as follows: φ = 50^o^18′59”N, λ = 18^o^46′18”E. There is also an automatic station of air quality monitoring located approximately 70 m from the IEE building and operated by the Regional Inspectorate for Environmental Protection (RIEP, http://powietrze.katowice.wios.gov.pl/stacje/stacja/5). The samples of PM_2.5_ for PBM determinations were taken at the RIEP station from a height of 4 m above the ground.

Złoty Potok is a village (1200 inhabitants) located in the north-eastern part of Silesian Province, about 20 km south-east of the city Częstochowa, and 45 km north-east of the Upper Silesian Agglomeration. The measurements of TGM and PBM concentrations were conducted at the air quality monitoring station (φ = 50^o^42′39”N, λ = 19^o^27′31”E) belonging to the RIEP. The gaseous and particulate sampling heads were located at about 4 m above the ground level. The station is situated within a typical rural landscape, and, being located in a relatively weakly polluted area, it serves as regional background measuring station for Silesian Province.

## 3. Measurement Methods

### 3.1. Measurements of Gas-Phase Mercury

At the RIEP’s station in Złoty Potok, TGM measurements were performed using Tekran 2537B (Tekran Inc., Toronto, ON, Canada), an automated analyzer of gaseous mercury. The measurement was based on the Hg amalgamation onto a gold cartridge (Au-trap), followed by thermal desorption in carrier gas and its detection as GEM via cold vapor atomic fluorescence spectrometry (CVAFS). The pre-filtered ambient air was passing through Au-trap to collect TGM. For continuous monitoring, the instrument utilized two Au-traps with alternating operation modes (collecting, desorbing, and chemical analysis) on a predefined time base (5min). It was calibrated daily using an internal mercury permeation source and checked periodically by manual injections of GEM from the Tekran 2505 calibration unit. 

Another measurement scheme was used at the IEE’s station in Zabrze for the Tekran 2537B/1130/1135 system. Coupled with various sample capture elements and selective desorption procedures, this system was used for quantitative analysis of speciated gaseous mercury, GEM, and GOM. The concentrations of TGM were determined indirectly as a sum of GEM and GOM. The air was pulled into the 1130 unit through an impactor designed to remove coarse particles. From the sample flowing over a KCl-coated denuder, the GOM was trapped and then fine particles (here: PM_2.5_) were separated within the 1135 unit. The remaining GEM was carried to the main module of Tekran 2537B for analysis. The GOM was accumulated for 1h while the GEM was pre-concentrated and detected every 5 min. After 1h sampling, GOM was thermally desorbed as GEM, transferred into the 2537B module and quantified by CVAFS. Speciated Hg measurements made by Tekran 2537B/1130/1135 systems have been described in detail elsewhere [24,25]. The manufacturer precision of the Tekran 2537B is 2% and the analytical detection limit of GEM is <0.75 pg for 5-min sampling intervals (7.5 l of ambient air) [26,27].

In this study, the results of short-term TGM measurements were averaged over 24 h on a given day. Thanks to this aggregating procedure, it was possible to directly compare TGM and PBM concentrations and the risk quotients calculated for these concentrations.

### 3.2. PM_2.5_ Sampling and Determination of Particulate Mercury

At the both sites, 24-hour samples of PM_2.5_ were collected by means of low volume Mikro PNS samplers (Umwelttechnik MCZ GmbH, Bad Nauheim, Germany) with jet/impactor PM_2.5_ sampling heads and automatic filter changers. The quartz fiber filters (Whatman QMA, minimum filtration capacity of 99.5%) were used. The samples were taken at a height of about 4 m above the ground level (Figure 1), according to the European reference method for gravimetric determination of PM_2.5_ mass concentration [28]. They were taken in series consisting of 14 24 h samples each (the sampler magazines capacity), and each accompanied by a field blank sample. During the whole measurement period, the number of n = 652 and n = 255 samples were collected in Złoty Potok and Zabrze, respectively. Immediately after sampling, the filters from Złoty Potok were conditioned and weighed in the RIEP’s laboratory in Częstochowa. Sections of these filters (and the weighing room and field blanks), each in a separate and sealed Petri dish, were transported using a thermo-container to the IEE’s laboratory in Zabrze to determine the mercury contents. The samples collected in Zabrze were conditioned and weighed in the IEE’s laboratory. To prevent Hg re-volatilization, all samples were stored in a freezer at −18 °C before the analyses. The procedures for conditioning, weighing, storage, and transport of the samples and of the blank sample preparation complied with the QA/QC procedures of the reference method for gravimetric measurements [28] and were described in detail in [29].

The Hg content was determined by applying cold-vapor atomic absorption spectrometry (CVAAS) to thermally decomposed PM_2.5_ samples. MA-2 analyzer (Nippon Instr. Co, Japan) was used. The section of exposed filter was placed in a boat and heated in the pipe furnace to 700 °C in a Hg-free air (carrier gas). The by-products of thermal decomposition (nitrogen oxides, sulfur dioxide, and halides) capable of interfering with Hg determination were eliminated by analytical additives and catalytic oxidation at 850 °C. Then, the decomposition products containing GEM, were passed through buffer solution (pH = 7), and, after drying, for improving the determination selectivity, they were passed through gold trap with amalgam formation. GEM, released upon rapid heating of the gold trap, were carried to detector, where the light absorbance of the mixture Hg/carrier gas was measured at the wavelength λ = 253.7 nm.

The analyzer was calibrated in the range of 0.1—6ng (R^2^ = 0.999). The calibration curve was obtained using calibration solutions. They were prepared based on the calibration standard (Inorganic Ventures, USA; certified Hg content: Hg 10 ppm). The method was validated using two standard reference materials: NIST1633b (Constituent Elements in Coal Fly Ash, certified Hg content: 0.1431 ± 0.0018 mg Hg kg^−1^) and NIST2583 (Trace Elements in Indoor Dust, certified Hg content: 1.56 ± 0.19 mg Hg kg^−1^). The mean standard recoveries (n = 10) were 90 ± 4% (in the range from 85 to 99%) for NIST 1633b and 96 ± 3% (from 73% to 102%) for NIST 2583. The limits of detection and quantification were determined by iteratively analyzing blank samples (n = 25) and they were 0.094 and 0.283 ng Hg, respectively. The standard deviation was 4.5% for an actual sample and 3.9% for SRM1633b. The repeatability, computed as a relative standard deviation from n = 25 measurements, was 4.5% for the actual sample and 3.9% for the NIST1633b sample [30]. 

## 4. Inhalation Exposure and Health Risk Assessment

The estimation of the potential non-carcinogenic health risk through inhalation of gaseous and particulate Hg was based on the methodology developed by the USA. EPA in the Risk Assessment Guidance for Superfund (RAGS) Part F - Supplemental Guidance for Inhalation Risk Assessment [31]. RAGS Part F provides the toxicity values derived by the EPA Integrated Risk Information System (IRIS) as so called reference concentrations (RfCs) instead of the earlier intake-based approach, used by the inhalation component of RAGS Part A [32]. According to the previous RAGS approach, the inhalation exposure, expressed in terms of a chronic daily air intake (I) [mg kg^−1^ day^−1^], was evaluated as a function of the contaminant concentration in the air (CA), inhalation rate (IR), body weight (BW), the exposure time (ET), exposure frequency (EF), and its duration (ED), as well as the averaging time (AT), in the following way:

I = CA × (IR/BW) × (ET × EF × ED)/AT,(1)

According to the Supplemental Guidance for Inhalation Risk Assessment of RAGS Part F [31], the inhalation exposure should be expressed as the exposure concentration EC [mg m^−3^] and calculated as follows:

EC = CA × (ET × EF × ED)/AT,(2)

where:

- CA [mg m^−3^] = 24 h concentrations of mercury species in the air (TGM or PBM);
- ET [hours day^−1^] = exposure time, here: 24 h day^−1^;
- EF [days year^−1^] = exposure frequency, here: 365 days year^−1^;
- ED [years] = exposure duration, here: lifespan 70 years;
- AT [hours] = averaging time calculated as the product of ED, EF and ET, here: (70 years × 365 days year^−1^ × 24 h day^−1^).

In order to estimate EC, an appropriate exposure scenario must be adopted. Despite the fact that, in a moderate climate, people spend most of their lifetime indoors (where Hg concentrations may be higher than those outside in ambient air [33]), we assumed that the inhabitants of Zabrze and Złoty Potok were constantly exposed to the atmospheric Hg concentrations. We assumed the most conservative exposure time, frequency, and its duration, lasting 24 h over the whole human lifespan. The uncertainties associated with such a choice are obvious and undisputed; however, in this study, we tried to estimate not the most likely exposure scenario but the worst case one, where exposure concentration is equal to Hg concentration in the air (EC = CA). When assessing EC, we did not include any age-dependent exposure variability. Inclusion of subsequent exposure variables into the risk model often causes the predictions to become more hypothetical.

The hazard quotient (HQ, unitless) reflecting the potential non-carcinogenic effect for the inhalation pathway was calculated separately for each Hg form with the following general equation:

HQ = EC/RfC,(3)

where RfC [mg m^−3^] = reference concentration for chronic inhalation exposure.

In this study we used the inhalation RfC=3 × 10^−4^ mg m^−3^ for elemental mercury, which is available in the IRIS database (https://cfpub.epa.gov/ncea/iris2/chemicalLanding.cfm?&substance_nmbr=370). The RfC for mercury is based on the assumption that thresholds exist for certain neurobehavioral effects. This value was set by EPA on the basis of some studies on workplace exposure with a high uncertainty factor [34]. We used the same RfC value for the both Hg forms, TGM and PBM. There is no separate inhalation RfC for particulate mercury or aerosol form of inorganic compounds, e.g., mercuric chloride, in the IRIS database (such RfC is under development).

For comparison purposes, we also used the proposal of revised reference concentration for elemental mercury (RfC_R_ = 0.7 × 10^−4^ mg m^−3^), presented by Lettmeier and co-authors [34] and based on the intensive cohort study.

The hazard index (HI, unitless) was applied to assess the cumulative risk of inhalation exposure to the both Hg forms. It was calculated as a sum of HQs:

HI = HQ_TGM_ + HQ_PBM_, (4)

HI or HQ values not exceeding 1 indicate no risk to health, while values above 1 indicate the risk of non-carcinogenic effects [32].

## 5. Results and Discussion

### 5.1. TGM and PBM Concentrations in the Air

**Figure 3:**
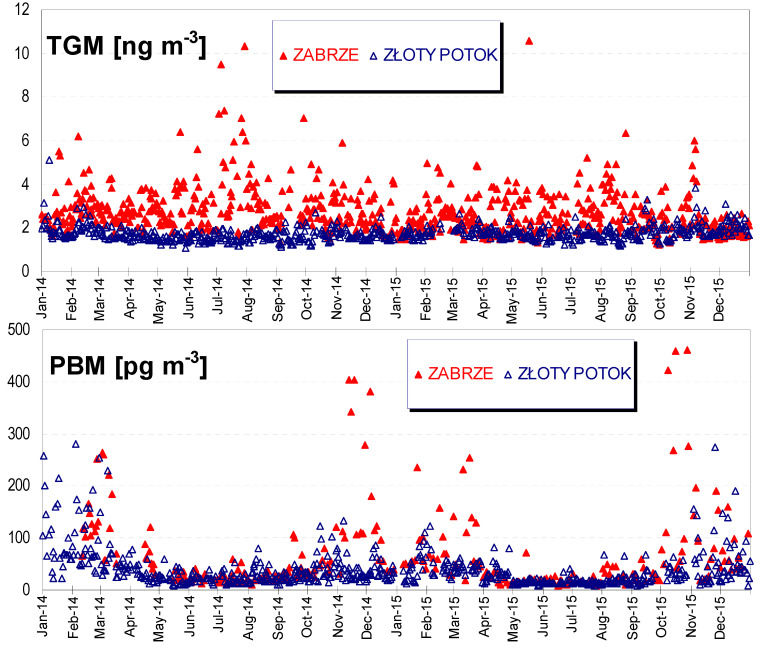
Daily concentrations of total gaseous mercury [TGM] and PM_2.5_-bound mercury [PBM] in Zabrze (urban background) and Złoty Potok (rural background), in the period of 2014–2015.

Time series of 24-h concentrations of TGM and PBM in Zabrze and Złoty Potok are shown in Figure 3.

In order to demonstrate statistically significant differences between the distributions of Hg concentrations at both locations, first the normality of each distribution was checked, and then the appropriate test was selected to verify the above hypothesis. The normality was checked by using the non-parametric Shapiro–Wilk test (Statistica StatSoft ver.10). Daily averaged TGM concentrations and 24 h PBM data, both the raw and log-transformed ones, showed non-normal distributions (*p* < 0.001). The ratio of skewness to its standard error was >2 for gaseous and particulate Hg which indicates that the data are highly right-skewed. For such asymmetric distribution, where arithmetic mean is typically greater than median, the latter was selected as a measure of a central tendency. The descriptive statistics for Hg data are presented in Table 1 and Table 2, with a distinction into summer season (a non-heating period from April to September) and winter (heating) season (the other 6 months). As a result of the U Mann–Whitney test, statistically significant differences between the distributions of Hg concentrations in Zabrze and Złoty Potok and their seasonality (summer vs. winter season) were confirmed (*p* < 0.05).

As can be seen in Figure 3, the higher TGM and PBM concentrations with clearly higher dispersion of data were obtained at the urban site in Zabrze. For this site, the overall median concentration of TGM and PBM was about 14 times higher than in Złoty Potok, and the standard deviations of TGM and PBM data were 45 and 24 times higher, respectively. To the best of our knowledge the concentrations of TGM and PBM in Zabrze were higher than anywhere else in Europe, excluding historical mercury mining areas in Spain, Slovenia, or Italy [35]. However, it should be noted that city locations of Hg monitoring stations are rather rare in Europe. Most of these stations are located in the coastal strip (cleaning effect of the sea breeze) or inland, in rural or remote sites. The nearest station of atmospheric Hg monitoring in Central Europe is German Waldhof (100km south-east of Hamburg), but this is a rural background site, more representative for our second station in Złoty Potok than for Zabrze. Selected results of TGM and PBM measurements in European locations and for comparison, in some urban sites of North America and Asia, are summarized in Table 3. The mean of TGM concentrations in Zabrze was lower than for Chinese cities, similar to those found in Seoul (South Korea), 2012–2013, and higher than reported by Mao and co-authors [36] for urban sites in Europe, North America, and Asia (median 2.1 ng m^−3^). 

**Figure 4:**
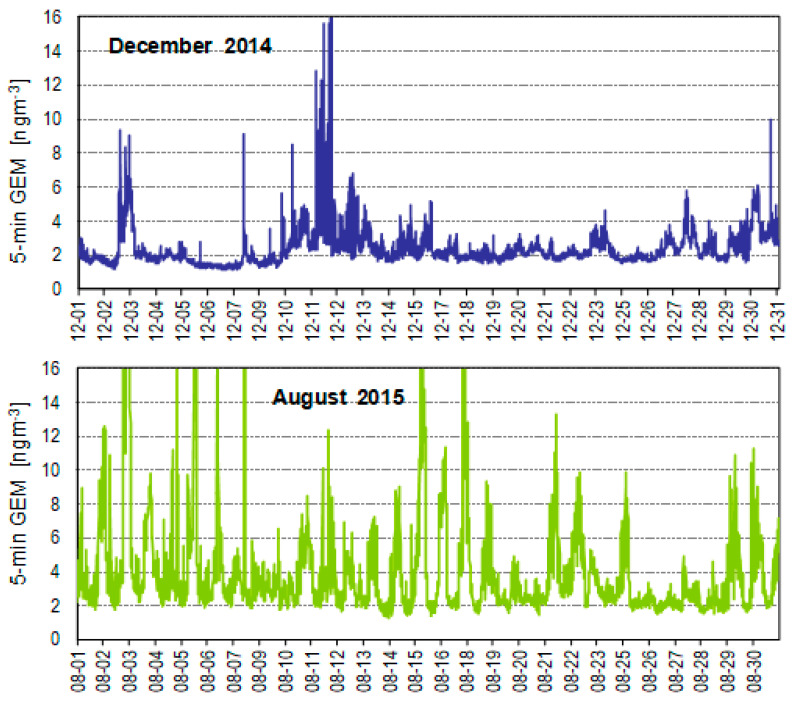
The concentrations of 5min GEM in December 2014 and August 2015 at the urban site in Zabrze (no diurnal pattern in winter and clear periodicity in summer with nocturnal maximum).

At the Zabrze location, higher TGM concentrations were recorded in summer than in winter season, in contrast to the seasonal variation of anthropogenic mercury emissions. This is an overlapping effect of meteorological and emission conditions, as well as the air inlet elevation (14 m a.g.l.). The elevated inlet allowed for recording the peak Hg concentrations in the summer season, caused by the local sources, including high emitters. In winter, the impact of higher emitters was limited due to the thermal inversion phenomenon, which prevents the pollution plumes from reaching the ground level. A similar seasonal ratio of TGM concentrations, resulting from the specified diurnal pattern in warmer months (with maximum at night, Figure 4), has been reported in other urban and industrial areas [36,55]. 

At the rural background station in Złoty Potok, a higher mean TGM concentration (by 12%) was obtained in the winter season, which is in line with the literature. According to [36], the predominant seasonal TGM/GEM trend at rural sites is the winter-to-early-spring maximum and summer minimum. Higher TGM concentrations in Złoty Potok in winter season were mainly driven by the anthropogenic mercury emissions for heating purposes (coal combustion throughout the region and local biomass burning) and limited mixing in the ground boundary layer, and to a lesser extent by poor GEM oxidation and less scavenging.

For both locations, Złoty Potok and Zabrze, very high PBM concentrations were obtained, well above the Central-European background (Waldhof, Germany) or levels at urban sites of Europe and North America, and closer to the values recorded in Chinese cities (Table 3). For comparison, Mao and co-authors [36] reported that the median PBM concentration was 10.0 pgm^−3^ at urban sites and 4.6 pgm^−3^ at rural sites, located on land, in non-polar regions of the Northern Hemisphere. Such high PBM concentrations are the result of the widespread use of coal burning in Silesia (and all over Poland) for heat and power generation [29,56]. The concentrations of PBM were proportional to high PM_2.5_ concentrations, which was 23 µgm^−3^ (median) in Zabrze and 16 µgm^−3^ in Złoty Potok during the whole analyzed period. The median PM_2.5_ concentration in Zabrze was three times higher in heating season than in summer. For Złoty Potok, this ratio amounted to 1.8. This was the main (and primary) reason for the statistically significant differences in PBM levels between the winter and summer seasons. Moreover, in the colder season the mercury contents in the PM_2.5_ samples were clearly higher than in summer. For Złoty Potok they amounted to 1.9 and 1.5 ng mg^−1^, respectively, and to 1.5 and 1.2 ng m^−1^ for Zabrze. Winter enrichment of PM_2.5_ samples with mercury by an additional 25%, compared to the summer season, was essentialy a secondary effect of lower temperature, higher relative humidity, and lower Hg vapor pressure. These factors led to a shift in the gas-particle partitioning towards the sorption of GOM on the solid particles.

### 5.2. Health Risk Resulting from Inhalation of TGM and PBM

**Figure 5:**
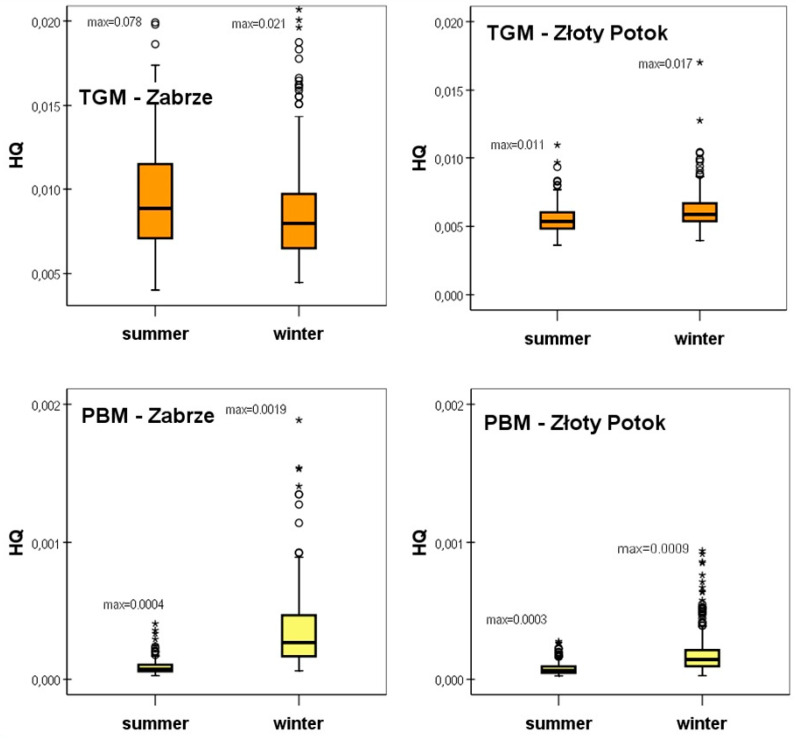
Seasonal distributions of hazard quotients (HQ) for total gaseous mercury (TGM) and PM_2.5_-bound mercury (PBM) in Zabrze (urban background) and Złoty Potok (rural background), in the period of 2014–2015.

The seasonal distributions of daily HQs, calculated for the current EPA’s RfC = 0.3 μgm^−3^, separately for TGM and PBM at the urban and rural station, are shown on the box plots in Figure 5. Each box includes the median (midline) and 25th and 75th percentiles (box edges). “Max” denotes the maximum value of HQs, which is representative rather for acute exposure and short-term (daily) risk. The measure of chronic exposure and the corresponding potential health risk is the median (or mean) of HQs. In our opinion the median values of HQs are more representative for right-skewed concentrations data; however, they are lower than the mean ones. Therefore, in Table 4 we compiled both the median and mean of HQs and HIs.

The median of the hazard quotient values in case of TGM concentrations in a more polluted urban area was 0.00828 throughout the entire analyzed period, therefore well below the HQ = 1 considered to pose a health risk.

Proportionally to the seasonal variations in TGM concentrations in Zabrze, the HQ value was 11% higher in summer season compared to winter one (median HQ was 0.00886 and 0.00798, respectively). The median of TGM HQs for the whole period in Złoty Potok amounted to approximately 68% of the corresponding HQs value in Zabrze. For the mean values, this proportion was only slightly lower and equal 62%. 

Regarding the median of PBM concentrations, the percentage share of HQ in HI in the whole analyzed period was 1.5% in Zabrze, and 1.6% in the case of Złoty Potok. For the mean values, these percentages were slightly higher (2.4 and 2.2%, respectively). The shares of HQ in HI for PBM increased on average by 1–2% in the winter season.They did not exceed 5% at both locations and did not significantly affect the level of the HI index.

Moreover, we calculated the HQs for TGM, PBM and the HIs resulting from the exposure to those species taking into account the revised reference concentration RfC_R_ = 0.07 μgm^−3^ [34], which is over four times lower than the current EPA’s RfC value (Table 5). In addition, in this case, the HI for the whole analyzed period was around 0.04 in Zabrze and did not exceed 0.03 in Złoty Potok, i.e., significantly below the value recommended by U.S. EPA as posing a health risk (HI = 1).

It is difficult to compare the obtained results of the assessment of hazard indexes with data from other places in the world, as we have not found similar exposure scenarios for calculating the cumulative inhalation risk resulting from TGM and PBM. Available literature evidences refer to either partial exposure to extremely high concentrations of gaseous mercury resulting from artisanal or small-scale gold production [57,58], partial exposure to aerosol mercury present in particles suspended in the air [59,60], or in settled dust [61,62]. Taking into account even one mercury intoxication pathway (e.g., by inhalation) and one form of mercury (e.g., PM-bound) the comparison of the HQ values in different places around the world is possible only when using similar exposure scenario. The differences in HQs levels, when using different exposure scenarios, can reach 3–4 orders of magnitude and are not proportional to Hg concentrations in the air. Large variations in health risks levels resulting from Hg exposure often meet in the literature mostly result from the lack of standardization regarding formulas used for calculating chronic daily air intake (I), formerly recommended by the U.S. EPA, among others, in relation to exposure estimation for soil compounds.

## 6. Conclusions

The paper presents results from a 2-year campaign of parallel measurements of gaseous mercury (TGM) and respiratory-related particulate mercury (PM_2.5_-bound Hg, PBM) collected in one of the European hot spot areas regarding mercury and PM emission levels. Measurements were carried out in Silesia, the most industrialized and densely populated region of Southern Poland in two locations differing in terms of emission characteristics. The obtained results and the nature of the concentration distribution for both forms of mercury were compared in locations representative for conditions of urban-industrial agglomeration and non-urban (rural) background. The recorded levels of TGM and PBM concentrations were higher than those from the Waldhof station (Germany), which can be considered as reference for the typical background levels characteristic for Central Europe. In the case of the urban-background station, the median and average values of TGM and PBM concentrations were higher than those observed in European or American locations of similar nature, and closer to the levels recorded in some urban stations in Asia (e.g., in Seoul, South Korea). These data indicate a significant impact of emissions of anthropogenic origin, generally associated with the widespread combustion of hard coal for the purposes of energy and heat production in power generation companies and municipal energy sector, but also in industry and individual home furnaces. EC exposure concentrations were determined by assuming the most unfavorable scenario of exposure of adult residents of Silesia to inhalation of the above-mentioned atmospheric mercury species (24 h exposure throughout the life span), followed by HQ hazard quotients and cumulative hazard index (HI) calculations. U.S. EPA methodology was used in the calculations. For the estimation of chronic exposure to mercury through inhalation, an appropriate reference concentration (RfC) is currently recommended by EPA. Calculations of HQs and HIs in both locations, representing different exposure potencies to airborne mercury in Silesia, were repeated for the revised reference concentration RfC_R_, proposed by Lettmeier and co-authors [34]. The obtained HIs, even after recalculation with the revised RfC_R_ value (4 times lower compared to RfC), were about 0.04 at the urban background site in Zabrze and below 0.03 at rural background site in Złoty Potok, throughout the entire measurement period. Therefore, the critical value of HI = 1 was not exceed in any case, including the sub-periods: summer/heating season. This means that relatively high concentrations of TGM and PBM, similar to those meet in Silesia, do not pose an inhalation health threat for residents. Estimating the values of the HI exposure indexes, it was found that, for PBM, the HQs constitute approximately 1% to 5% of the total HIs values. Thus, despite high PBM concentrations found in this study, compared to other places in Europe or North America, the proportion of PM-bound mercury is rather marginal in creating the cumulative Hg-dependent inhalation risk. Looking for literature confirmation of the correctness of the HQs and HIs calculations made in our work, it was noticed that even taking into account only single intoxication pathway and one form of mercury, it is strictly impossible to compare HQs calculated for different places in the world. The differences in HQs levels reach 3–4 orders of magnitude and do not reflect the actual levels of Hg occurrence in the environment. They result from the use of various calculation formulas, including those recommended by U.S. EPA. For the need of comparability, there exists a need for harmonization of the HQ estimation approach able to include variation resulting from multiple sources (multi-environment) of Hg origin and multiple absorption paths (multi-path).

## Figures and Tables

**Table 1:** Descriptive statistics of the measurement results (24 h concentrations) of total gaseous mercury (TGM) and PM_2.5_-bound mercury (PBM) at the urban background station in Zabrze, 2014–2015.

Statistical Parameter	Summer Season	Winter Season	Whole Period
TGM			
Min – Max [ng m^−3^]	1.21–23.26	1.34–6.21	1.21–23.26
Mean ± SD [ng m^−3^]	3.06 ± 2.02	2.57 ± 0.87	2.81 ± 1.56
Median [ng m^−3^]	2.66	2.40	2.48
95 percentile [ng m^−3^]	5.17	4.26	4.76
Number of measurements	331	351	682
PBM			
Min – Max [pg m^−3^]	7.64–121.06	18.78–565.67	7.64–565.67
Mean ± SD [pg m^−3^]	27.68 ± 18.62	118.74 ± 106.58	69.81 ± 86.53
Median [pg m^−3^]	21.58	80.21	37.87
95 percentile [pg m^−3^]	62.40	385.15	255.89
Number of measurements	137	118	255

**Table 2:** Descriptive statistics of the measurement results (24 h concentrations) of total gaseous mercury (TGM) and PM_2.5_-bound mercury (PBM) at the rural background station in Złoty Potok, 2014–2015.

Statistical Parameter	Summer Season	Winter Season	Whole Period
TGM			
Min–Max [ng m^−3^]	1.09–3.28	1.19–5.11	1.0–5.11
Mean ± SD [ng m^−3^]	1.65 ± 0.27	1.85 ± 0.39	1.74 ± 0.35
Median [ng m^−3^]	1.60	1.76	1.69
95 percentile [ng m^−3^]	2.10	2.51	2.31
Number of measurements	358	326	684
PBM			
Min–Max [pg m^−3^]	7.44–82.49	7.67–281.71	7.44–281.71
Mean ± SD [pg m^−3^]	22.84 ± 12.76	54.77 ± 44.27	38.48 ± 36.01
Median [pg m^−3^]	19.19	42.68	27.82
95 percentile [pg m^−3^]	49.40	150.57	102.21
Number of measurements	330	317	647

**Table 3:** Comparison of TGM and PBM concentrations in selected sites in Europe, North America, and Asia.

Country, Site of Measurements	Site Description	Period	TGM/GEM/ [ng m^−3^] *	PBM [pg m^−3^] *	Reference
Europe					
Germany, Waldhof	rural (Central- European background)	2009–2011	/GEM/		[37]
			1.61	6.30	
			1.1–3.1	0.4–110	
UK (Scotland), Auchencorth Moss	rural	2009–2011	/GEM/		[38]
			1.40 ± 0.19	3.11 ± 5.34	
UK (England: London, Manchester Sheffield; Ireland: Belfast; Scotland: Motherwell; Wales: Cardiff, Swansea)	urban	2004–2013	2.07 ± 0.03 (manual measurement)	-	[39]
Ireland, Mace Head (North Atlantic)	regional background, coastal site	1996–2009	1.65 ± 0.13	-	[40]
		2012–2015	1.48 1.48 ± 0.13	-	[41]
Sweden, Råö (Baltic Sea)	rural, coastal site	2012–2015	/GEM/		[41]
			1.41	2.21	
			1.42 ± 0.20	3.6 ± 4.5	
Germany, Zingst (Baltic Sea)	rural, coastal site	1998–2004	1.66	-	[42]
Sweden, Göteborg (Baltic Sea)	urban, coastal site	2005	1.96 ± 0.38	2.53 ± 4.09	[43]
Portugal, Porto (Atlantic Ocean)	suburban coastal site	2008–2014	1.93 (mean) 0.51–67.9	-	[44]
Poland, Zabrze (Silesia)	urban	2014–2015	2.48 2.81 ± 1.56	37.87 69.81 ± 86.53	this study
Poland, Złoty Potok (Silesia)	rural, regional background	2014–2015	1.69 1.74 ± 0.35	27.82 38.48 ± 36.01	this study
North America					
USA, Reno NV	urban	2002–2005	2.3 (mean) 2.1 (median) 0.9–8.6		[45]
		2007–2009	2.0 ± 0.7	7.0 ± 7.0	[46]
USA, Birmingham AL, New York City, Rochester NY, Salt Lake City UT	urban (*AMNet*)	2009–2011	/GEM/		[47]
			1.51	4.97	
			1.65 ± 1.36	8.46 ± 29.05	
			0.4–237.7	0–3687	
Canada, Halifax (North Atlantic)	urban, costal site	2010–2011	/GEM/		[48]
			1.7 ± 1.0	2.3 ± 3.1	
Asia					
China (Southeast Ch.), Xiamen	urban	2012–2013	/GEM/		[49]
			3.50 ± 1.21	174.4 ± 280.6	
China (Eastern Ch.), Jinan	urban	2015–2016	4.91 ± 3.66	451.9 ± 433.4	[50]
China, Beijing	urban	2016–2017	/GEM/		[51]
			4.70 ± 3.53	85.18 ± 95.34	
China (Northwest Ch.), Lanzhou	urban	2016–2017	4.48 ± 2.32	-	[52]
South Korea, Seoul	urban	2006–2009	3.72 ± 2.96	13.4 ± 12.0	[53]
			0.19–149.84	2.1–64.3	
South Korea, Seoul	urban	2012–2013	2.34 ± 0.73	-	[54]
Northern Hemisphere background			1.5–1.7	-	[5]

* TGM/GEM/and PBM concentrations – as: Median; Mean ± SD or Minimum–Maximum; *AMNet* - Atmospheric mercury network.

**Table 4:** The median and mean of hazard quotients (HQ) and hazard indexes (HI) for TGM and PBM concentrations in Zabrze and Złoty Potok (2014–2015) and the actual EPA’s reference concentration RfC = 0.3 μgm^−3^.

Statistical Parameter	HQ for TGM		HQ for PBM		HI for TGM and PBM	
	Zabrze	Złoty Potok	Zabrze	Złoty Potok	Zabrze	Złoty Potok
Summer season						
Median [unitless]	0.00886	0.00535	0.00007	0.00006	0.00893	0.00541
Mean [unitless]	0.01020	0.00548	0.00009	0.00008	0.01029	0.00556
Winter season						
Median [unitless]	0.00798	0.00587	0.00027	0.00014	0.00825	0.00601
Mean [unitless]	0.00856	0.00616	0.00040	0.00018	0.00896	0.00634
Whole period						
Median [unitless]	0.00828	0.00562	0.00013	0.00009	0.00840	0.00571
Mean [unitless]	0.00936	0.00581	0.00023	0.00013	0.00959	0.00593

**Table 5:** The median and mean of hazard quotients (HQ) and hazard indexes (HI) for TGM and PBM concentrations in Zabrze and Złoty Potok (2014–2015) and the proposal of revised EPA’s reference concentration RfC_R_ = 0.07μgm^−3^.

Statistical Parameter	HQ for TGM		HQ for PBM		HI for TGM and PBM	
	Zabrze	Złoty Potok	Zabrze	Złoty Potok	Zabrze	Złoty Potok
Summer season						
Median [unitless]	0.03798	0.02293	0.00031	0.00027	0.03829	0.02320
Mean [unitless]	0.04371	0.02350	0.00040	0.00033	0.04411	0.02383
Winter season						
Median [unitless]	0.03422	0.02516	0.00115	0.00061	0.03536	0.02577
Mean [unitless]	0.03669	0.02640	0.00170	0.00078	0.03838	0.02718
Whole period						
Median [unitless]	0.03548	0.02409	0.00054	0.00040	0.03602	0.02449
Mean [unitless]	0.04010	0.02488	0.00100	0.00055	0.04109	0.02543
